# Homologous or Heterologous COVID-19 Booster Regimens Significantly Impact Sero-Neutralization of SARS-CoV-2 Virus and Its Variants

**DOI:** 10.3390/vaccines10081321

**Published:** 2022-08-15

**Authors:** Rome Buathong, Taweewun Hunsawong, Supaporn Wacharapluesadee, Suriya Guharat, Ratthapat Jirapipatt, Sasiprapa Ninwattana, Nattakarn Thippamom, Anusara Jitsatja, Anthony R. Jones, Kamonthip Rungrojchareonkit, Jindarat Lohachanakul, Rungarun Suthangkornkul, Kedsara Tayong, Chonticha Klungthong, Stefan Fernandez, Opass Putcharoen

**Affiliations:** 1 Department of Disease Control, Ministry of Public Health, Nonthaburi 11000, Thailand; 2 Department of Virology, Armed Force Research Institute of Medical Sciences, Bangkok 10400, Thailand; 3 Thai Red Cross Emerging Infectious Diseases Clinical Center, King Chulalongkorn Memorial Hospital, Bangkok 10330, Thailand; 4 Samut Sakhon Provincial Health Office, Ministry of Public Health, Nonthaburi 11000, Thailand; 5 Faculty of Medicine, Chulalongkorn University, Bangkok 10330, Thailand

**Keywords:** COVID-19, vaccine, booster, variants of concern, neutralization

## Abstract

We determined the levels of neutralizing antibodies against the SARS-CoV-2 ancestral strain, Delta and Omicron variants of concern (VOCs), in 125 healthcare workers who received CoronaVac as their primary vaccination and later received either a single ChAdOx1 or a combi-nation of two consecutive boosters using either two ChAdOx1 doses or a ChAdOx1 or BNT162b2 as the primary and second boosters, respectively, or two doses of BNT162b2. The titers 12 weeks after primary vaccination were inadequate to neutralize all strains. After a single ChAdOx1 booster, the levels of neutralization at Day 30 varied significantly, with only a small proportion of participants developing neutralizing titers against Omicron at Day 7 and 30. The two doses of ChAdOx1 as the booster induced the lowest activity. A combination ChAdOx1 and BNT162b2 induced greater neutralization than by two doses of ChAdOx1. Two doses of BNT162b2 as the booster had the maximal activity against Omicron VOC.

## 1. Introduction

Starting in 2020, the availability of COVID-19 vaccines has marked the most significant effort to control the coronavirus disease (COVID-19) and reduce severe acute respiratory syndrome coronavirus (SARS-CoV-2) infections [1]. Evidence demonstrates the effectiveness of COVID-19 vaccines to prevent infection and the severity of COVID-19 [2,3]. However, vaccine-induced immunity wanes over time and boosting is required for continued protection against emerging SARS-CoV-2 variants [4,5]. We have previously reported immunity after primary vaccination with CoronaVac (Sinovac) and ChAdOx1 nCoV-19 (Vaxzevria, Oxford-AstraZeneca) vaccines [6,7]. With the emergence of the Alpha, Beta, Delta, Gamma, and Omicron variants of concern (VOCs), as recognized by the World Health Organization (WHO), vaccine boosters are essential to maintain adequate immunity. Studies into the extent and durability of vaccine-induced neutralizing antibody levels are very important to identify optimal boosting strategies. Approaches for booster application are diverse due to the availability of different vaccine platforms in each country. Homologous or heterologous COVID-19 prime-boost vaccinations have been reported to improve humoral and cellular immune responses. A homologous third dose of CoronaVac (an inactivated SARS-CoV-2 vaccine, Sinovac Life Science) demonstrated increased immune responses against SARS-CoV-2; however, immunity was lower when compared to heterologous boosting with BNT162b2 [8]. The Beta, Delta, and Omicron VOCs have shown the ability to evade immune responses induced by vaccination or prior infection [9,10,11]. Previous studies have shown a decrease in booster-derived immunity protection against Delta and Omicron VOCs [12,13]. Additional studies are needed to dissect antibody levels after a second booster. A 4th dose of BNT162b2 (Pfizer–BioNTech) or mRNA-1273 (Moderna) have been shown to induce IgG antibodies against SARS-CoV-2 receptor-binding domain (RBD) and increase neutralizing antibody titers to levels higher than in unboosted individuals. In addition, the rates of SARS-CoV-2 infection and severe COVID-19 were lower after a fourth dose of BNT162b vaccine, underscoring the importance of boosters against Omicron infection, especially among those at high risk for severe COVID-19 [14]. CoronaVac, an inactivated COVID-19 vaccine, is widely used in many countries, which have adopted this vaccine for primary prevention. Little data exist describing booster-derived immunogenicity against VOCs such as Delta and Omicron in recipients of CoronaVac as the primary vaccination. In this study, we determined the levels of neutralizing antibodies by microneutralization assays against the ancestral strain of SARS-CoV-2 and the Delta and Omicron VOCs in COVID-19 vaccinated healthcare workers (HCWs) who received CoronaVac as their primary vaccination and later received either a single ChAdOx1 booster or a combination of two consecutive boosters using either two ChAdOx1 doses or a ChAdOx1 or BNT162b2 as the primary and second boosters, respectively, or 2 doses of BNT162b2.

## 2. Materials and Methods

### 2.1. Cell Line and Virus

Vero E6, a green monkey kidney epithelial cell line was obtained from ATCC. Cells were grown in Eagle’s minimum essential medium (EMEM, Invitrogen, Waltham, MA, USA) supplement with 5% heat-inactivated fetal bovine serum (HIFBS, Invitrogen, USA), 1% L-glutamine, 1% P&S, 40 µg/mL gentamicin, and 0.25 µg/mL fungizone, at 35 ± 2 °C, 5% CO_2_ incubator. One-day-old cells were used for measuring of neutralizing antibody by microneutralization assay.

SARS-CoV-2 viruses, ancestral lineage (isolate Hong Kong/VM20001061/2020, NR-52282), was obtained through BEI Resources (NIAID, Manassas, VA, USA). The Delta B.1.617.2 (hCoV-19/Thailand/CU-A21287-NT/2021, GISAID Accession ID; EPI_ISL_2510689) and Omicron BA.1 (hCoV-19/Thailand/CU-A211051-NPS/2022, GISAID Accession ID; EPI_ISL_14175998) variants were isolated from clinical specimens collected at King Chulalongkorn Memorial Hospital. All isolates were propagated in Vero E6 cells to generate sufficient titers (100TCID50) for the microneutralization assay. All isolates were quantitated by tissue culture infectious dose 50 (TCID50) using the Reed-Muench method based on eight replicates per titration.

### 2.2. Microneutralization Assay

A microneutralization (MN) assay was used to determine neutralizing (NT) antibodies against SARS-CoV-2 viruses including the ancestral strain, B.1.617.2 (Delta) and BA.1 (Omicron) variants [15]. All procedures were performed in a BSL-3 laboratory, following a standard neutralization assay using cytopathic effect (CPE)-based colorimetric read-outs. Each assay included eight cell control (CC) and virus control (VC) wells. Clotted blood was collected and centrifuged at 3000 RPM for 15 min and stored at −80 °C until testing. Serum samples from the same patient were tested on the same batch. Serum samples were diluted with 2% HIFBS/EMEM media (Invitrogen, USA) at 1:10 dilutions before heat-inactivation at 56 °C for 30 min. An equal volume of diluted serum was separately incubated with 100 TCID50 of SARS-CoV-2 ancestral strain, and with B.1.617.2 (Delta), and BA.1 (Omicron) variants in 37 °C, 5% CO_2_ incubator for 1 h. The 100 µL of serum-virus mixture (final serum dilution is 1:20) was inoculated into duplicate wells of VeroE6 cells in 96-well plates and incubated at 37 °C, 5% CO_2_ for 5 days before staining with 0.02% neutral red (Sigma, St. Louis, MO, USA) in 1X PBS (Invitrogen). After an additional incubation at RT for 1 h, lysis solution was added before measuring OD at 540 nm. Percentage of virus infectivity in VC and samples were calculated based on OD of CC, Infectivity of VC (%) = (OD of CC–OD of VC) × 100 and Infectivity of sample (%) = (OD of CC − OD of sample) × 100. The percentage of inhibition was calculated using the following formula, Inhibition (%) = 100 − [(100 × Infectivity of sample)/Infectivity of VC]. Percentage of inhibition at equal or higher than 50% (≥50% inhibition) is considered as positive cut off for neutralizing antibodies against the SARS-CoV-2 strain or a variant.

### 2.3. Statistical Analysis

Data analysis was performed using R program Version 4.0.2 and GraphPad version 9.0 (GraphPad Software, Inc., San Diego, CA, USA). Paired *t*-test, Mann–Whitney U test or Wilcoxon test was used to compare the mean of % inhibition obtained against different SARS-CoV-2 variants and the differences between base line and post-boost sera. A *p*-value < 0.05 was considered statistically significant.

## 3. Results

### 3.1. Demographic Data of Participants

**Figure 1:**
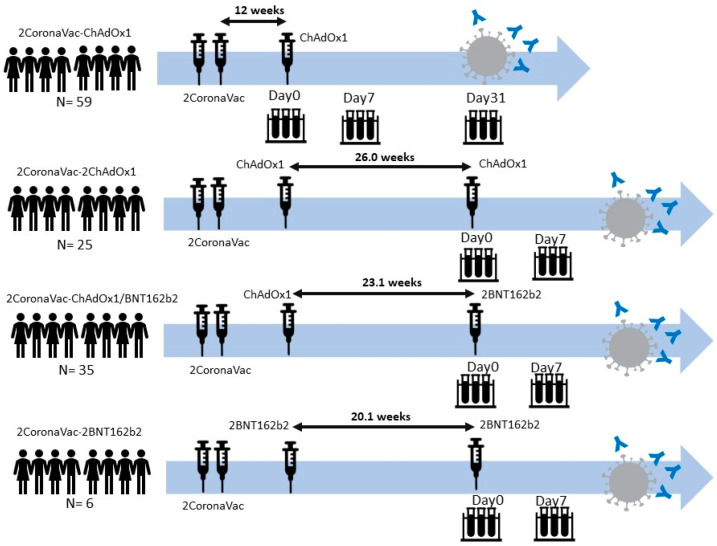
Vaccination and booster distribution and schedules.

This is an observational study in which people with different booster schemes were retrospectively included, and their booster-schemes were not planned. A total of 125 health care workers were enrolled in this study, all of which completed primary COVID-19 vaccination with CoronaVac (two doses). Table 1 and Table 2 list the demographic characteristics of all subjects. All these HCWs were highly aware of their infection risk. They had to follow the surveillance protocol such as completing a daily questionnaire to screen for COVID-19 symptoms and exposure risk. Any HCWs who had any symptoms or risk factors were tested for SARS-CoV-2 infection using the RT-PCR technique. The PCR-positive subject was excluded from the study. Subjects were divided into four distinct groups (Figure 1). Fifty-nine (59, 2CoronaVac-1ChAdOx1, Table 1) subjects received a single ChAdOx1 booster 12 weeks after their primary vaccination. Blood samples were collected at day 0, 1 week, and 4 weeks after receiving the booster dose. Blood samples were used to measure neutralizing antibodies. This group did not receive a second booster during this study. Twenty-five (25, 2CoronaVac-2ChAdOx1, Table 2) participants received two consecutive ChAdOx1 boosters at approximately 12 weeks post primary vaccination and 182 days (Median 26.0 weeks, IQR: 21.4–26.5) after the first booster, respectively. Blood samples were collected at day 0 and 1 week after the second booster. Thirty-five (35, 2CoronaVac-ChAdOx1/BNT162b2, Table 2) subjects received a ChAdOx1 booster (12 weeks after primary vaccinations) and a BNT162b2 as a second booster 162 days (Median 23.1 weeks, IQR: 21.4–26.6) afterwards. As before, blood samples were collected at day 0 and 1 week after the second booster. Six (6, 2CoronaVac-2BNT162b2, Table 2) subjects received two consecutive BNT162b2 boosters, approximately 12 weeks after the primary vaccinations and 141 days (Median 20.1 weeks, IQR: 20.1–20.2) after the primary booster, respectively. Again, blood samples were collected at day 0 and 1 week after the second booster. The median time between the primary vaccination and the first booster for all subjects was 14.57 (Median, IQR: 14.00–17.3) weeks and the median time between the first and the second booster was 23.43 (Median, IQR: 21.4–26.5) weeks. The outline of subjects’ vaccine/booster distribution and schedule is detailed in Figure 1. Boosters for HCWs in Thailand was recommended soon after the detection of VOCs. Booster vaccinations were selected based on HWCs’ preferences.

### 3.2. Neutralizing Antibodies against SARS-CoV-2 Ancestral Strain, Delta, and Omicron VOCs

**Figure 2:**
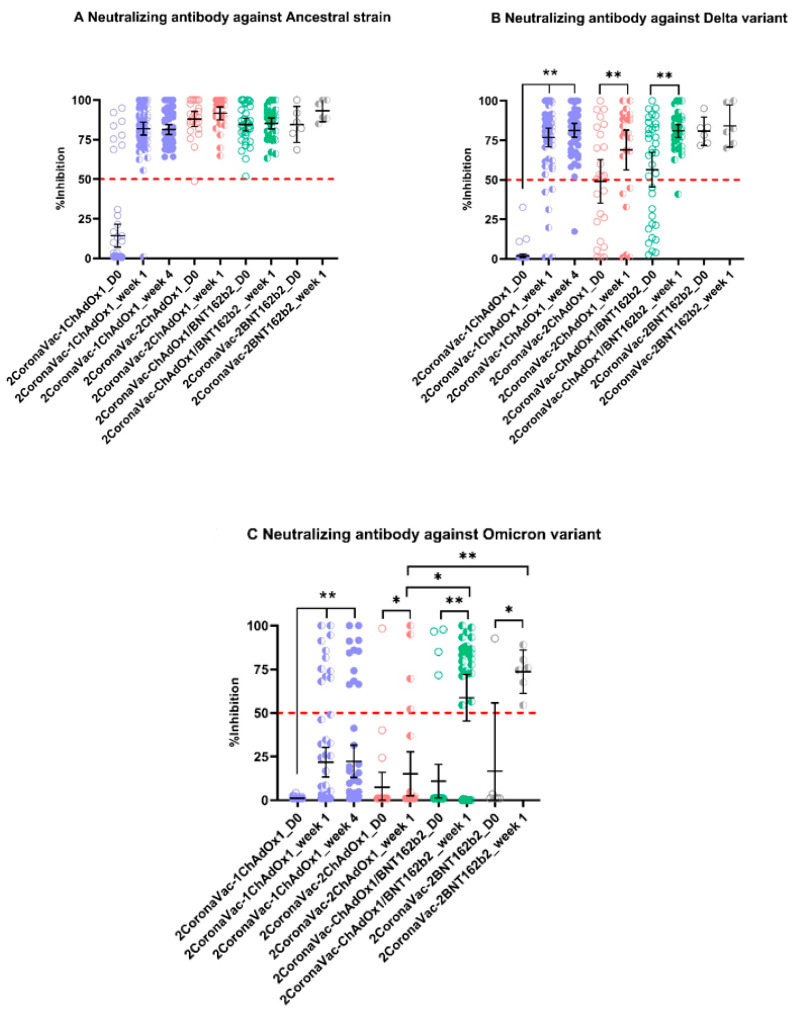
Neutralizing (NT) antibodies against SARS-CoV-2 Ancestral strain (**A**), Delta (**B**), and Omicron (**C**) VOCs. A “*” indicates significance of *p* < 0.05. A “**” indicates significance of *p* < 0.0001.

**Figure 3:**
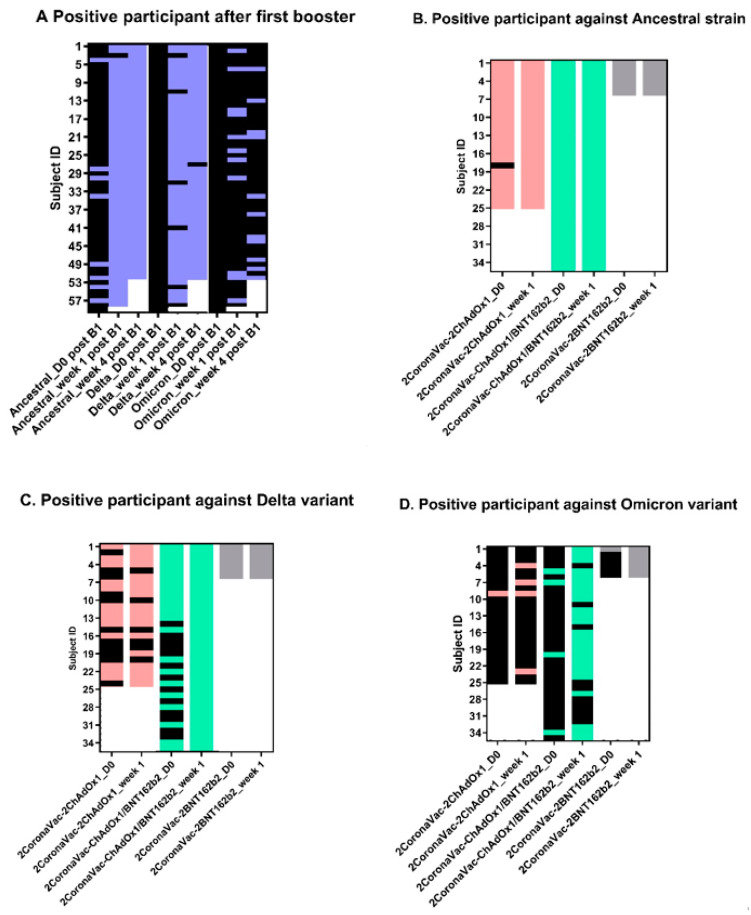
Proportion of participants with microneutralization titers at equal or higher than 50% (≥50%) against Ancestral, Delta, and Omicron variant at day 0 (D0) and post first or second boost-er. A group of subjects ((**A**), *n* = 59) received a first 1ChAdOx1 booster at day 0 (D0 post B1). Blood samples were collected at day 0, week 1, and week 4 for microneutralization assay. Purple color indicates individual subjects who are positive for microneutralization and black color indicates negative microneutralization. Groups which received a second booster received 2CoronaVac-2ChAdOx1 (*n* = 25), or 2CoronaVac-ChA-dOx1/BNT162b2 (*n =* 36) or 2CoronaVac-2BNT162b2 (*n =* 6) at day0 (D0). Blood was collected on day 0 and week 1 post second booster and used for microneutralization assays against ancestral strain (**B**), Delta variant (**C**), and Omicron variant (**D**). Rose, green, and grey colors indicate individual subjects who are positive for microneutralization in the 2CoronaVac-2ChAdOx1 (*n* = 25), 2CoronaVac-ChA-dOx1/BNT162b2 (*n* = 35), and 2CoronaVac-2BNT162b2 (*n* = 6) groups, respectively. Black color indicates negative microneutralization.

We determined neutralizing (NT) antibodies (both as mean percentage inhibition and NT positivity rate) against the SARS-CoV-2 ancestral strain, Delta and Omicron VOCs, for all groups (Figure 2A–C and Figure 3). A sample was reported as positive for NT antibodies if achieving viral neutralization higher than 50%. When measured against the SARS-CoV-2 ancestral strain, only 13.6% of subjects in the 2CoronaVac-1ChAdOx1group were NT positive at the time of booster dose (Day 0, Figure 2A) and a mean percentage inhibition (MI) of 14.3% (95% CI: 7.1–21.5%). However, 98.5% of subjects were NT positive at week 1 (*p* < 0.0001) with an MI of 81.9% (95% CI: 77.8–86.0%) and a 100% at week 4 post-booster, with an MI of 81.3% (95% CI: 78.2–84.4%). Ninety-six percent (96%) of the 2CoronaVac-2ChAdOx1 subjects who received a second ChAdx1 booster dose were NT positive at the time of their second booster (day 0) with an MI of 88.1% (95% CI: 82.2–92.9%). All (100%) were NT positive at week 1 post second booster and an MI of 91.6% (95% CI: 87.5–95.7%). Similarly, 100% of the subjects in the 2CoronaVac-ChAdOx1/BNT162b2 group and in the 2CoronaVac-2BNT162b2 group were NT positive against the ancestral strain at day 0 (MI of 84.5%, 95% CI: 80.5–88.6%; and MI of 84.6%, 95% CI: 73.2–95.9%, respectively) and at 1 week (MI of 85.2%, 95% CI: 81.8–88.7%; and MI of 93.3%, 95% CI: 86.3–100.3%, respectively) after their second booster dose. Figure 2B shows NT antibody titers against the Delta VOC for all groups. In the 2CoronaVac-1ChAdOx1group, only 8.5% of subjects were NT positive against the Delta VOC at day 0 post-booster, with a MI of 1.9% (95% CI: 0.7–3.1%), but the percentage increased significantly (*p* < 0.0001) to 91.5% at week 1 post-booster with an MI of 76.8% (95% CI: 70.9–82.7%) and again to 98.1% at week 4 post-booster with an MI of 81.3% (95% CI: 76.9–85.7%). The rates of NT positivity against the Delta VOC among the 2CoronaVac-2ChAdOx1 subjects increased from 56% with an MI of 49% (95% CI: 35.3–62.7%) at Day 0 post second booster to 76% with an MI of 70.4% (95% CI: 58.9–81.9%, *p* = 0.005) at week 1 post second booster. Among the 2CoronaVac-ChAdOx1/BNT162b2 subjects, % rates increased from 62.9% with an MI of 56.4% (95% CI: 45.5–67.3%) at Day 0 to 97% with an MI of 80.9% (95% CI: 76.8–85.1%) at week 1 post second booster. One hundred percent (100%) of all subjects in the 2CoronaVac-2BNT162b2 group were NT positives against the Delta VOC at Day 0 (MI of 80.7%, 95% CI: 71.8–89.7%) and week 1 (MI of 84.1%, 95% CI: 70.7–97.5%) post second booster. Figure 2C shows NT antibody titers against the Omicron VOC for all groups. In general, NT rates and titers against Omicron VOC were relatively low for all groups and timepoints. NT positivity rate for subjects in the 2CoronaVac-1ChAdOx1at day 0 post-booster was 0% with an MI of 1.2% (95% CI: 1.0–1.3%), which increased significantly (*p* < 0.0001) at week 1 to 18.6% with an MI of 21.8% (95% CI: 13.3–30.3%) and to 21.2% with an MI of 22.2% (95% CI: 12.9–31.5%) at week 4. Only 4% of subjects in the 2CoronaVac-2ChAdOx1 group were NT positive against the Omicron VOCs at Day 0 post second booster, with an MI of 7.4% (95% CI: 0–16.0%). NT positive rates increased to 16% at week 1 with an MI of 15.1% (95% CI: 2.6–27.7%). NT positivity rate among subjects in the 2CoronaVac-ChAdOx1/BNT162b2 group went from 11.4% with an MI of 10.0% 95% CI: 0.2–19.8%) at Day 0 to 71.4% with an MI of 58.7% (95% CI: 45.4–72.0%) at week 1. NT positivity rate among subjects in the 2CoronaVac-2BNT162b2 increased from 16% with an MI of 16.0% (95% CI: 0–55.4%) at Day 0 to 100% with a MI of 73.6% (95% CI: 61.2–86.1%) at week 1. Subjects in the 2CoronaVac-ChAdOx1/BNT162b2 and 2CoronaVac-2BNT162b2 groups had significant higher MI against Omicron VOC than the CoronaVac(x2)/ChAdOx1(x2) group at week 1 post second booster (*p* = 0.034 and *p* < 0.0001, respectively).

## 4. Discussion

This study was conducted between June 2021 and February 2022. The CoronaVac vaccine has been used as the primary vaccination in many countries, including Thailand, but not exclusively. Other vaccination options in Thailand include homologous vaccination with ChAdOx1 or BNT162b2 (Pfizer-BioNTech) and heterologous prime boost by using CoronaVac-ChAdOx1. During the early phase of vaccine distribution in Thailand, there was the limited availability of mRNA vaccines (BNT162b2), with availability only beginning in June 2021. Healthcare workers and populations at risk for hospitalization and death from COVID-19 were prioritized early during vaccination. The first variant found in Thailand was characterized by the presence of the D614G mutation. The Alpha, Delta, and Omicron VOCs emerged in Thailand in February, April 2021, and February 2022, respectively. As vaccine-induced antibody responses wane overtime, vaccine boosters are thus recommended to maintain optimal protection against infection and severe COVID-19 disease [16]. Similarly, continued vaccine-induced immune responses are required for protection against immune evasion by emerging VOCs [17]. Participants in this study are health care workers who had received CoronaVac as the primary vaccination followed different booster regimens, as determined by the participant’s own preference, workplace policy, availability of boosters, and emergence of new variants of SARS-CoV-2. The primary vaccination program was implemented during the Alpha VOC predominance in Thailand. With the emergence of the Delta VOC, a booster program was recommended by the Thai Ministry of Public Health and aimed to restore immunity against severe COVID-19 caused by the Delta VOC. With the introduction of the Omicron VOC in February 2022, an additional second booster was recommended, especially for healthcare workers and individuals at high risk for severe disease.

From our findings, neutralizing titers 12 weeks after primary vaccination with the inactivated CoronaVac COVID-19 vaccine are low and inadequate to neutralize the ancestral strain of SARS-CoV-2 and the Delta and BA.1 Omicron VOCs. This is evident by the low rate of NT positivity and low mean inhibitory titers found in the serum of CoronaVac recipients at the time of their (first) ChAdOx1 booster (Figure 2A–C). The rate of NT positivity and mean inhibitory titers increased rapidly by Day 7 after a single ChAdOx1 booster and remained so through Day 30. However, the levels of neutralization at Day 30 varied significantly, with moderate to low levels against the Delta and Omicron VOCs, with only a small proportion of participants developing neutralizing titers against Omicron at Day 7 and 30 after the ChAdOx1 booster. Although not directly measured from the 2CoronaVac-1ChAdOx1group (the only group receiving a single ChAdOx1 booster dose), we can infer (from the 2CoronaVac-2ChAdOx1 and 2CoronaVac-ChAdOx1/BNT162b2 groups) that the single ChAdOx1 booster-derived neutralizing titers against SARS-CoV-2 ancestral strain remained unchanged for at least 160–180 days, as Day 0 blood sample in these two groups is collected 160–180 days post their first ChAdOx1 booster. The same is not the case for neutralizing titers against the Delta and Omicron VOCs, which seemed to have eroded further by 160–180 days after the ChAdOx1 booster. The 2CoronaVac-2BNT162b2 group, although small (*n* = 6) allows to compare the humoral immunogenicity of the first BNT162b boost and compare it to the ChAdx1 booster, above. At 141 days post BNT162b boost (and at the time that the second BNT162b boost was applied), all subjects remained NT positive against the Delta VOC, in contrast to the first ChAdx1 boost received in the other groups. There were no differences in neutralization against the ancestral strain and Omicron VOC. 

In the groups that received a second boost dose, this was applied between 141 and 182 days after the first one. The effect of a second booster was rapid (measured at 7 days) and broad. Mean neutralizing against the ancestral strain did not change significantly, and this may have been caused by the already high and sustained titers generated by previous boosts. However, mean neutralization increased significantly against the Delta VOC when a second ChAdOx1 (*p* = 0.0078) or BNT162b2 (*p* < 0.001) booster was used. Similarly, against the Omicron VOC, a second ChAdOx1 or BNT162b2 booster induced rapid neutralization. The data shows that a second BNT162b2 booster generated significantly (*p* = 0.034) higher mean neutralizing than a second ChAdOx1 booster, suggesting that BNT162b2 might be more suitable for induction of antibody responses against the Omicron VOC. Ultimately, in this study, the data show that the use of the BNT162b2 as both first and second booster generated the strongest responses against all strain and variants. An additional dose of BNT162b2 after two doses of CoronaVac as the primary vaccination showed a significant increase in the levels of neutralizing antibodies against Omicron in a study conducted in Brazil [18]. Our data support the use of BNT162b2 as a second booster that might maintain adequate neutralization against Omicron. 

In summary, these data indicate that people previously vaccinated with an inactivated CoronaVac SARS-CoV-2 vaccine can benefit from a single ChAdOx1 booster as early as one week after administration as this booster rapidly induced neutralizing antibodies against both the ancestral strain, Delta VOC and, less so, against the Omicron VOC. The challenges caused by the arrival of new SARS-CoV-2 variants make two-dose booster regimen likely necessary to induce or maintain optimal levels of neutralizing antibodies against these new variants, such as the Omicron VOC. The findings suggested that two doses of ChAdOx1 as the booster induced the lowest activity against Delta and Omicron variants. A combination of a booster regimen by ChAdOx1 and BNT162b2 induced greater neutralization against Delta and Omicron than by two doses of ChAdOx1, but the levels of neutralization against Omicron were moderate. Two doses of BNT162b2 as the booster had the maximal activity against Omicron compared to other booster regimens. The immune response against Omicron increased rapidly at 1 week after the fourth booster.

There are some limitations of this study, including the lack of a nonrandomized design, neutralization experiments in terms of co-infection, and the limited number of participants. Additionally, the data generated in this study were based on an in vitro experiment only which, although it can be used as a predictive tool, lacks the ability to replicate the overall process inside human body or in an in vivo system. Further studies looking at the kinetics and durability of humoral immunity after single or repeated homologous and heterologous vaccine boosters will be required to document kinetics of humoral immune protection.

## 5. Conclusions

Vaccines to prevent SARS-CoV-2 infections are considered the most important approach for curbing the COVID-19 pandemic. Several COVID-19 vaccine platforms are available globally. Booster doses are necessary to enhance immunity against SARS-CoV-2 variants. Our study suggests that rapid and robust immunogenicity can be obtained with heterologous booster regimens against the Omicron variant. A first dose of ChAdOx1 elicits at least some degree of neutralizing antibodies against the Omicron VOC but these levels of neutralization decreased rapidly after 12 weeks post booster. Additional boosters are required. However, boosters, their type, and frequency have to be carefully calibrated as neutralizing antibodies differ in their longevity and capacity to neutralize current and future circulating variants. Although limited by the number of subjects, our data suggest that using mRNA vaccines, at least as part of a booster regimen, provides higher neutralizing anti-bodies against new VOCs.

## Figures and Tables

**Table 1:** Demographic data of participants who received primary vaccination by CoronaVac and ChAdOx1 as the first booster.

Number of Participants	59
Age, median (years)	40
IQR	32.5–52.5
20–30 years	11
31–40 years	19
41–50 years	13
51–60 years	15
61–70 years	1
BMI, median	22.2
IQR	20.9–26.3
Range	16.1–33.2
Duration between 2CoronaVac and ChAdOx1, median (weeks)	14.1

**Table 2:** Demographic data of participants who had received primary vaccination by CoronaVac and then had the first and second booster by 2ChAdOx1 or ChAdOx1/BNT162b2 or 2BNT162b2.

Characteristics	2CoronaVac-2ChAdOx1	2CoronaVac-ChA-dOx1/BNT162b2	2CoronaVac-2BNT162b2	*p*-Value
Number of participants	25	35	6	
Age, median (years)	42	35	33	0.370
IQR	36–54	2.5–49	29.5–44	
Range	22–62	23–60	28–57	
Age group, *n*				
20–30 years	5	11	2	
31–40 years	6	9	2	
41–50 years	6	7	1	
51–60 years	7	8	1	
61–70 years	1	0	0	
Sex (% Male)	32.0	42.9	66.7	0.283
BMI, median	21.5	24.0	25.4	0.489
IQR	19.4–27.5	20.9–25.91	24.15–26.36	
Range	16.0–33.2	16.37–30.32	21.97–27.51	
Duration between 2CoronaVac and the first booster, median (weeks)	14.5	16.7	19.3	0.0212
Duration between the first and the second booster, median (weeks)	26.0	23.1	20.1	0.0047
IQR	21.4–26.5	21.4–26.6	20.1–20.2	
Range	21.4–27.4	13.0–28.0	16.0–20.2	

## Data Availability

The supporting data for the findings of this study are available from the corresponding author upon reasonable request.
